# Exploring paramedic professional identity

**DOI:** 10.29045/14784726.2023.12.8.3.42

**Published:** 2023-12-01

**Authors:** Lawrence Hill, Georgette Eaton

**Affiliations:** University of East Anglia ORCID iD https://orcid.org/0000-0002-9147-0934; University of Oxford; London Ambulance Service NHS Trust ORCID iD: http://orcid.org/0000-0001-9421-2845

**Keywords:** philosophy of practice, professional identity

## Abstract

The notion of a paramedic (as a title protected in law) has recently entered its third decade, but the history of the paramedic is considerably older than that. Who are we as a profession? What does it mean to be a paramedic? What makes us who we are? These intriguing and yet seldom asked philosophical questions are at the heart of this article, which is intended to provoke discussion and serve as a foundation for further inquiry into questions of identity and philosophy in paramedicine.

Literature pertaining to paramedic professional identity was explored and contextualised within current paramedic practice. Although the overall picture is complex, four key areas for discussion emerged: the history of the paramedic profession; role diversity; the influence of ambulance work; and the education and training of paramedics. The influence of ambulance work permeates all areas, suggesting that it is central to the development of paramedic professional identity. This discussion article is an exploration of the unique contexts and experiences that are associated with the process of being and becoming for paramedics.

## Introduction

There has been much debate about paramedic identity in recent times, particularly as paramedics in the United Kingdom continue to expand their scope of practice into what have previously been considered non-traditional paramedic occupational spaces, with commensurate changes in skills and knowledge. Such professional development and increasing professional freedom is at risk of causing an identity crisis, a kind of Kierkegaardian existential vertigo (Kierkegaard, 1980) and perhaps a sense of imposter syndrome.

The existing body of literature pertaining to paramedic professional identity is limited (Johnston & Acker, 2016; Johnston & Bilton, 2020; O’Meara, 2011) and, like any question of identity, is nebulous and complex (Burke & Stets, 2009). While there exists some exploratory work on identity in sociological theory for paramedic research (Johnston & Acker, 2016) and questions of professional identity in paramedic students (Johnston & Bilton, 2020), there is little by way of inquiry into how paramedics conceptualise their own identity as professionals, apart from a conference presentation by Hill and Eaton (2023). At a time of unprecedented growth and development of the profession, a discussion of the ‘deeper philosophical concern regarding the essence of paramedic practice’ (O’Meara, 2011, p. 57) seems overdue.

## What is professional identity?

An identity is the set of meanings that in combination define who we are, and our place in society. When one is an occupant of a particular role in society, or member of a specific group, this influences one’s identity and confers a sense of belonging and meaning (Burke & Stets, 2009).

A professional identity is defined as ‘the attitudes, values, knowledge, beliefs and skills that are shared with others within a professional group’ (Adams et al., 2006, p. 56) and operates both at an organisational and individual level (Cascón-Pereira & Hallier, 2012). Professional identity facilitates gathering and understanding the knowledge and skills associated with professional work, as well as the values and dispositions of professions (Sutherland & Markauskaite, 2012). Tsakissiris and Grant-Smith (2021) claim that the relationship between occupation/profession and identities explains the significance of ‘what we do’ for our sense of ‘who we are’, and that it creates a psychological attachment between an individual to a particular profession.

As a profession, paramedicine requires a discipline-specific knowledge (knowing) and enacting that knowledge (doing) for the benefit of other people within a purposeful and informed framework – both technical and ethical (O’Meara, 2011). These concepts (doing and knowing) are relatively easy to espouse, but only provide a partial picture of what defines a professional beyond their competencies and skills. The development of a professional identity is as much about ‘becoming’ and ‘being’ as it is about ‘knowing’ and ‘doing’. In this sense, professional identity is a continuous process of professional socialisation that is formed by various means including through professional education and during placement (Ashby et al., 2016).

It appears that paramedic professional identity emerges from something more profound than merely the doing and knowing aspects of practice. Undergoing a process of professional socialisation and then embodying a role (becoming and being) includes considering the historical reason for the development of the profession, discussing its purpose and exploring its point of distinction from other health professions.

## What is a paramedic?

In 2019, the College of Paramedics (UK) attempted to define the profession, stating:

A paramedic works autonomously as a generalist clinician across a range of healthcare settings, usually in emergency, primary or urgent care. They may also specialise in clinical practice, education, leadership or research (Eaton, 2019a, p. 1).

This definition accurately describes aspects of what a paramedic *does* but does not attempt to discern the existential nature of *being* a paramedic – which, in its defence, it did not intend to do. The dictionary definition above, as well as the standards of proficiency for paramedics (Health and Care Professions Council, 2023), describe an omnicompetent practitioner in a way that is devoid of any sense of deeper meaning, in the same way that a definition of water gives no impression of wetness, despite describing important and irrefutable properties of water.

And so, the ‘is’ of the question ‘what is a paramedic’ depends on one’s philosophical orientation, but grasping questions of identity requires stepping beyond definitions of ‘doing’ and ‘knowing’ to include aspects of ‘being’ and ‘becoming’, as these are inseparable from the process of identity formation and maintenance and the Heideggerian sense of being-in-the-world for paramedics – a state of living with a highly meaningful orientation (Heidegger, 1962).

Their definition goes on to discuss what education and skills paramedics have that distinguish them from other healthcare practitioners as well as hinting at aspects of ‘becoming’: ‘Paramedics are educated and trained to make decisions in complex and high-pressure situations in unfamiliar and often unpredictable environments’ (Eaton, 2019a, p. 1), which provides a description that is more closely connected with praxis and may resonate more strongly with the experiences of paramedics than the accurate but necessarily limited definition of the ‘generalist clinician’ above.

More recently, Williams et al. (2021, p. 3568) used a Delphi study methodology to provide a definition of what paramedicine is:

Paramedicine is a domain of practice and health profession that specialises across a range of settings including, but not limited to, emergency and primary care. Paramedics work in a variety of clinical settings such as emergency medical services, ambulance services, hospitals and clinics as well as non-clinical roles, such as education, leadership, public health and research. Paramedics possess complex knowledge and skills, a broad scope of practice and are an essential part of the healthcare system. Depending on location, paramedics may practice under medical direction or independently, often in unscheduled, unpredictable or dynamic settings.

Their generalist definition conflates paramedicine with paramedics themselves, which is interesting from an existential perspective, and suggests that they consider there is something vital about the sense of ‘being’ an individual paramedic that transcends what they know and where they practise (which is what constitutes the majority of their definition).

This concept of a generalist exists within the professional standards for paramedics. The Health and Care Professions Council’s (HCPC’s) standards that serve to regulate the education and practice of paramedics are, more or less, the same for all 15 of the allied health professions (AHPs). The Health and Care Professions Council’s ‘Standards of conduct, performance and ethics’ (2018a), ‘Standards of continuing professional development’ (2018b) and ‘Standards of education and training’ (2018c) are all generic standards common to all AHPs. Only the HCPC ‘Standards of proficiency’ (Health and Care Professions Council, 2023) are differentiated, and even then, large parts of these standards include generic standards of knowledge and skills common to all AHPs.

**Figure 1:**
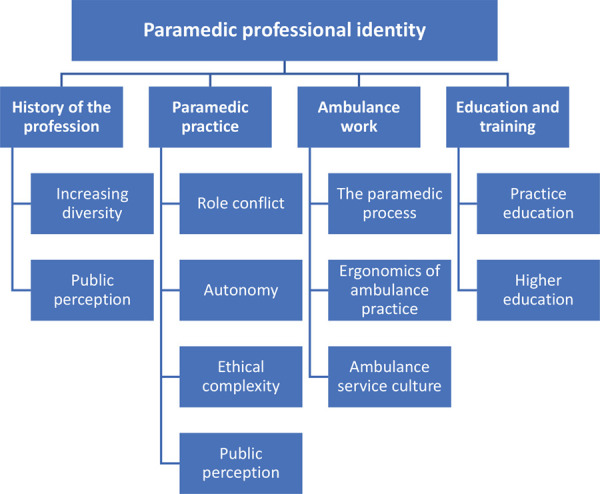
Figure 1. Concepts associated with paramedic professional identity.

What is unsaid by these standards, but well known to any paramedic, is that paramedicine is a complex set of diverse, connected and interdependent fields, which require practitioner adaptability to facilitate the solving of novel problems in dynamic situations. We consider this within four broad categories (see Figure 1) in the remainder of this article, synthesised into a coherent dialogue in an attempt to address O’Meara’s (2011, p. 57) ‘deeper philosophical concern regarding the essence of paramedic practice’.

## History of the paramedic profession

It is 20 years since the advent of professional registration for paramedics in the United Kingdom, and 25 years since the first paramedics entered the academy at the University of Hertfordshire to undertake the first paramedic-centric higher education programme (Newton & Hodge, 2012). These are both key features of the professionalisation of any role (Johnston & Acker, 2016; McCann et al., 2013), and yet paramedic professional identity is not defined simply by its status as a registered profession or the things that paramedics simply know and do (O’Meara, 2011).

The values and ethos of the modern paramedic profession stem from the unique function that paramedicine first performed in civil society following the Crusades of the 11th century. Much like nursing had been associated with care because of its historical associations with nunneries, paramedicine became associated with rescuing or saving those who had been injured or were sick and in need of definitive care and/or cure. In the United Kingdom, paramedics as we recognise them today emerged initially from the ‘ambulance men’ (and they were *mostly* men) of the 1970s in the south of England (Baskett et al., 1976, p. 377). Clinical training of these ambulance workers focused on techniques to assess and treat those presenting with coronary artery disease or in cardiac arrest (Briggs et al., 1976) and traumatic injuries (Baskett et al., 1976) due to road traffic collisions – which were highly prevalent in the 1960s and 1970s (Keep & Rutherford, 2013).

These clinicians were able to systematically bring emergency care, including aspects of advanced life support (e.g. advanced airway management and defibrillation), into the community for the first time. Fast forward more than 50 years, and although advanced emergency care remains central to paramedic practice in the 21st century, the nature of practice has evolved significantly since the 1970s, in line with the moving needs of the NHS and the changing nature of call type from ‘life threatening’ to more ‘treat-and-refer’, social problems, mental health issues and end-of-life care (Association of Ambulance Chief Executives, 2011; Department of Health, 2005; NHS, 2014, 2019).

The modern definition of a paramedic (Eaton, 2019a, p. 1) alludes to the importance of paramedicine’s beginnings in the ambulance service but also reflects the contemporary praxis, and argues that the key point in differentiation for paramedics from other healthcare practitioners today stems from those beginnings:

The core role of the paramedic still requires that we are capable of operating as a generalist, in the sense that we deliver care to patients of all ages, who may present with a broad range of conditions, with varying complexity and within a wide range of environments. All this, autonomously, from the point of registration.

This point of distinction continues to exist and remains relevant when discussing paramedic identity. But it creates some tensions for paramedic identity when paramedics move away from ambulance practice to elsewhere, and in so doing alter their role – and therefore the experiences that they have – which begs the rhetorical question: To what extent is a paramedic, who does not work on an ambulance, still a paramedic?

Moreover, paramedics now have a different educational background (College of Paramedics, 2019), a different gender profile compared to the ambulance men of the 1970s (Johnston & Acker, 2016) and different career aspirations (Proctor, 2019). They are no longer confined to the ambulance environment (College of Paramedics, 2023), working in a very wide range of services and settings (College of Paramedics, 2020) while retaining their professional title.

Although the paramedic profession emerges from a traditional emphasis on emergency care, the work of paramedics on ambulances (and indeed in all areas where paramedics can now be found working) is very different from the 1970s in the south of England. Paramedicine continues to evolve and deliver more nuanced and diverse functions. Looking from the vantage point of the 21st century, the paramedic of the 1970s appears to have been a well-defined role with a clear sense of purpose. While the paramedic of the 2020s shares much of their professional DNA with the UK paramedics of the 1970s, the profession has evolved into a different species. This diversification and successful integration with other areas of work showcases the potential of the paramedic profession but also raises questions relating to professional identity – as social agents we are partly a product of our environment (Burke & Stets, 2009). Professional registration, higher educational standards and integration into wider healthcare systems are all indirectly contributing to the formation of a professional identity (O’Meara, 2011), but so too are the direct experiences of occupying a paramedic role and, crucially, the environment in which one fulfils that role (Campeau, 2008).

## Paramedic practice

When individuals occupy social roles (such as teacher, firefighter or paramedic) and identify with them, they develop a role identity. Role identity theory describes a process by which individuals develop identity through interaction with their environment or community of practice (Stryker, 1980). When considering role identity within paramedic practice, concepts including role conflict, autonomy, ethical complexity and public perception are evident.

## Role conflict

Historically, it has been reasonably clear what a paramedic’s role was: the resuscitation of the acutely sick and injured. However, with the contemporary expectations of employers, a proliferation of non-traditional roles, a shift in focus from vocational training to a graduate-entry profession and changing expectations of the public, opportunities for role conflict are emerging beyond the expected heterogeneity of any professional role identity and stretching the notion of the traditional ambulance paramedic role.

Although it is already recognised that role identity is not homogenous for paramedics (Donnelly et al., 2015), an individual who enacts roles that are in conflict with each other, or are against their value systems, may experience a form of stress called ‘role conflict’ (Brief et al., 1979). Another form of role conflict occurs when an individual is in a role where the values, performance levels and behaviours are not clearly understood. This can be extremely difficult territory for paramedic students and new registrants to negotiate (Johnston & Bilton, 2020; Lazarsfeld-Jensen, 2019).

Role stress can also occur when the expectations of the individual do not reflect the work that they are doing, or are expected to do by their employers (Wanous et al., 1992). Indeed, there may be opportunities for conflict between expectation and reality, as well as dissonance between how university lecturers expect students to practise and how students perceive the way that practice is done ‘on the road’ (Lazarsfeld-Jensen, 2019). A further source of incongruence for paramedics is explored by Mausz et al. (2022), who describe a tension between an espoused and able-to-enact paramedic role identity. In terms of the experience of these paramedics (and paramedics and paramedic students more widely), there is a gap between ‘work-as-imagined’ and ‘work-as-done’ (Hollnagel et al., 2015), because there is little that can prepare people for the reality of ambulance work – this results in a significant opportunity for role dissonance. The paramedics in Mausz et al.’s (2022) study who were able to recalibrate their sense of self and understanding of their role in light of the work-as-done were better able to reconcile the dissonance and reduce the likelihood of emotional and psychological distress, disability and lost time from work.

An emergency medical services role-identity scale has previously been developed to bring some order to the myriad roles occupied by paramedics within the ambulance setting (Donnelly et al., 2015). This 71-item identity scale consists of four identified domains of caregiving, thrill seeking, capacity and duty. Such a scale recognises that paramedics are capable of reconciling an internal dualism of ‘rescuer’ traits with those of ‘care giver’, but that this may provide a challenge for them. While their study only explored ambulance roles, as the range of environments where paramedics can be found working diversifies, it is likely that further unpredicted role conflicts may emerge. Indeed, this has been found as paramedics transition from the ambulance service to work in primary care (Eaton et al., 2021) and also into higher education (Munro et al., 2018). Conversely, paramedics moving from an ambulance environment into a Canadian multi-professional emergency department environment have indicated that they believe the work aligns with their professional identity (Whalen et al., 2018).

In addition to Stryker’s (1980) theoretical stance that role identity emerges from an individual’s exposure to and immersion within their environment and community of practice, Archer’s (1995) realist ‘morphogenetic’ approach to the development of role identity suggests a more balanced relationship between social structures and individual agency. This is consistent with the idea that paramedics are actively renegotiating their professional identity to align with the environment in which they work. So, although there is some conflict within the ambulance paramedic role, it also appears that paramedic identity may transcend the ambulance environment in an adapted form. In fact, Whalen et al. (2018) suggest it may be possible to discern ambulance identity from paramedic professional identity, enabling a closer examination of the latter.

Paramedics inhabit multiple and possibly dissonant role identities simultaneously, for example carer and rescuer (Donnelly et al., 2015), professional and blue-collar-quasi-professional (McCann et al., 2013), but also are required to balance conflicting and dissonant ethical complexities, for example involving autonomy and non-maleficence (Sandman & Nordmark, 2006). From an identity theory perspective, this dissonance in itself introduces a kind of existential stress or role tension that may subtly promote the shifting of identity over time, as one adjusts one’s identity to suit a social/working environment (Brief et al., 1979; Burke & Stets, 2009). Frequent exposure to these dissonant social pressures may explain why paramedics appear to develop traits such as adaptability, and the capacity to operate with autonomy in grey areas, which are aspects of professional identity that have facilitated the permeation of paramedics into wider healthcare areas.

## Autonomy

Autonomy is a key feature of contemporary UK paramedic practice (Capsey, 2010; College of Paramedics, 2021; Health and Care Professions Council, 2018a, 2023) and has been a feature of ambulance work since the 1970s. In their scoping review, Reed et al. (2019) found that paramedics value their autonomy and assert it on a daily basis as they undertake their paramedic roles. In a similar vein, Burges Watson et al. (2012) identified that paramedics were concerned about the potential loss of professional autonomy as a result of participating in research protocols, where strict research criteria were perceived as a threat to their judgements about diagnosis. Involvement in research, and the resultant perceived loss of autonomy, therefore had the potential to both limit and enhance professional identity (Burges Watson et al., 2012). Due to the multiplicity of areas of work for ambulance paramedics (emergency care, obstetrics, trauma, minor injuries and illness, mental health, etc.), this sense of autonomy may be felt more intensely by paramedics and have a greater influence on their professional identity, which they may then carry with them into other work roles.

## Ethical complexity

Ethical dissonance is also a common feature of the paramedic role (Murray et al., 2018; Sandman & Nordmark, 2006). Paramedics are often forced to make decisions in an attempt to resolve problems that have no clear solution, but where each option in some way impedes the wishes, liberty or indeed ongoing life of individuals and their loved ones. This introduces the risk of ‘moral injury’ (Murray et al., 2018), which can be acquired for a variety of reasons, including situations where one is required to take actions that are contrary to one’s moral and ethical persuasion.

Another example of ethical complexity, particularly for students, is with the anecdotes and stories that can become a defining socio-cultural narrative in the shaping of professional identity and culture (Lazarsfeld-Jensen, 2014). Previous research has found that paramedic students emulate the values expressed by their practice educators in order to succeed (Eaton, 2019b), and so it is likely that stories have similar implications for professional socialisation. These stories may be shocking, irreverent and overtly dissonant with a student’s sense of what appropriate humour is (Christopher, 2015), but they may also be an emergent property of the inherently dissonant, ethically challenging and highly autonomous role of the paramedic in ambulance practice – where many formative experiences occur for paramedics. Ultimately, paramedics use stories to find meaning and even humour in bizarre and tragicomic circumstances. Although this has professional implications and risks, which need to be considered and weighed by paramedics, humour and a broader street-level philosophy that partially emerges from reconciling ethical complexity serve paramedics as coping strategies and form part of their identity.

## Ambulance work

The long-established image that paramedics work within an ambulance service is based on a delivery model focused on responding to a call, initiating treatment and transporting the patient to definitive care. However, the transformation of paramedicine over the last decade (associated with the higher education threshold entry to registration and the changing demands of the ambulance services) has transformed the profession, with opportunities for paramedics to play a greater role as diagnosticians. Being generalist clinicians, paramedics retain a unique position as one of the most accessible healthcare providers in the healthcare service – a position that cannot be replicated in other clinical settings. With such a valuable disposition, it is no wonder that paramedics are increasingly sought after to fill workforce gaps away from the out-of-hospital arena. Ambulance work may perhaps be the single greatest contributor to the professional bearing and identity that gives paramedics such broad utility.

Ambulance work is distinctive from other health disciplines, due to the highly variable and unpredictable work conditions (Capsey, 2010). The ‘paramedic process’ which identifies the unique features of ambulance practice for paramedics, postulated by Carter and Thompson (2015), goes some way to characterise and define what it means to undertake the role of an ambulance paramedic. Many features of the paramedic role are unique to the ambulance practice discipline. Most notably, the random and unpredictable environment in which they perform their duties as ambulance paramedics, but also the sequence with which these processes occur. The unique selling point of paramedics (and a feature at the core of their professional identity) does not lie in any specific clinical skill but in how and where those skills are applied (Capsey, 2010). Paramedics are unique among health professionals in that they apply clinical skills in a dynamic, potentially hostile and highly variable environment involving objective risks, limited resources, scant information and unpredictable individuals. Campeau (2008) argues that far from this need for control of space being circumstantial to paramedic practice, it is part of the very definition of paramedic professional identity. The ability to control space, and provide effective care despite the circumstances, is central to effective ambulance paramedic practice and seems to be important in the formation of paramedic professional identity. It is quite possible that the paramedic process, learned through education and experience in ambulance practice, coupled with the nature of the working environment itself, leaves an indelible imprint on paramedics, influencing their professional identity long after they leave the ambulance setting. This may in fact be what gives paramedics abundant utility outside of the ambulance setting.

If Campeau’s (2008) argument that on-scene management is critical to the formation of paramedics’ professional identity is true for the positive characteristics of a paramedic’s identity and capabilities, then it may also be true that ambulance work is responsible for some of the more negative consequences such as burnout, moral injury, occupational stress, role conflict and traumatic stress. Burnout features heavily in the literature relating to paramedic professional identity (Deniz et al., 2016; Donnelly et al., 2015; Granter et al., 2019; McCann et al., 2013), with occupational stress also being frequently mentioned (Burford et al., 2014; Granter et al., 2019; Harrison, 2019; Johnston & Acker, 2016; Lazarsfeld-Jensen, 2014, 2019). It is not possible to ignore these negative aspects of practice and their impact on identity. There are a number of tensions identified in the literature that may contribute to burnout, and not all of them are obvious.

One source of tension is that ambulance work has a tendency to lack continuity, which can lead to a sense that work is not rewarding (Whalen et al., 2018). Ambulance work is the antithesis of continuity. Campeau’s (2008) ‘space control theory’ and Carter and Thompson’s (2015) ‘paramedic process’ illuminate just how little continuity there is and how paramedics have to create continuity and security in the space around them to enable them to deliver effective care in austere environments. Although there are undeniable problems and challenges posed by the ergonomics of ambulance practice, experienced paramedics anecdotally have an assured, quietly confident and outwardly calm bearing even when faced with novel complex and time-sensitive problems. This may be a consequence of a familiarity and comfort with a lack of continuity in clinical practice – leading to features of paramedic professional identity.

A second, less than obvious, source of tension related to the question of paramedic identity is that of ‘carer/rescuer dissonance’. Lazarsfeld-Jensen (2014) observes that the expectations of students joining the profession are seldom met by the reality of paramedic practice. Popular media and portrayals of paramedic practice seldom capture the messy reality of practice: the reality of working in an ambulance or response car; the poverty and deprivation of patients; waiting times associated with delivering care; moral and ethical dilemmas; the complexity of decision making; vicarious trauma as well as actual exposure to violence and aggression; and the moral injury incurred by exposure to the above (Murray et al., 2018). Although stereotyping paramedics is common, their identity should not be assumed – and yet through no fault of their own this is what is happening to new students. Paramedic students may have a dream of what paramedicine will be like, but they are far removed from the reality of practice until they begin their studies – because access to the reality of ambulance work is not possible until embarking on a degree-level programme of study (Donaghy, 2010).

A third, subtle source of tension for paramedics relating to the ergonomics of ambulance work stems from the ‘command and control’ nature of ambulance dispatch, which Carter and Thompson (2015) identify as an essential part of the paramedic process. Paramedics working in the ambulance service are dispatched to attend calls, and in many cases are directed what to do by employer protocols that may at times be at odds with national guidelines. Yet they must be fully accountable for their actions on arrival. This introduces a dissonance between autonomy and non-autonomy. This external locus of control may contribute to the formation of a ‘blue-collar identity’ (McCann et al., 2013) through the exertion of institutional control over workers preventing them from being truly autonomous practitioners in that they lack the ‘professional authority’ identified by Greenwood (1957) as a hallmark of a profession.

A final source of tension may be itself an emergent property of the complexity of ambulance work – ambulance service culture. Organisational culture is a complex and amorphous concept, subject to various local contingencies, and is difficult to define. Ambulance service culture can be challenging, and possibly detrimental to paramedic students as they are socialised into ambulance service practice and assume a professional identity.

## Education and training

University education undoubtedly has an important role in the development of the professional identity of paramedics (Johnston & Bilton, 2020), partly because a significant part of the university experience for paramedic students will be practice education. In the United Kingdom, paramedic programmes are regulated by the HCPC, which stipulates the threshold requirements of paramedic education (Health and Care Professions Council, 2018c). In addition to the statutory regulation provided by the HCPC, the College of Paramedics provides its own paramedic education curriculum guidance document, scope of practice policy and career framework (College of Paramedics, 2019, 2021, 2023). When these documents are considered alongside the Quality Assurance Agency’s (2019) ‘Subject benchmark statement: Paramedics’, what emerges are similar but divergent expectations of what a paramedic should be able to do.

The College of Paramedics (2019) curriculum guidance places emphasis on a range of clinical placements but also stresses the centrality of ambulance placement for the development of safe and effective paramedics. In addition to the intended learning outcomes from these placement experiences, paramedic students will be working out how they can master the hidden curriculum and be accepted into the workplace and begin to assimilate the ambulance identity (Granter et al., 2019; McCann et al., 2013; Wankhade et al., 2018). Indeed, part of the hidden curriculum for paramedic students is the development of professional capital. According to McCann et al. (2013), this depends on socialisation into a ‘blue-collar’ professional workplace, which immediately brings into tension the expectations placed by higher education on students with their subsequent experiences as a new professional, not to mention the expectations the student brings with them about the role. There is opportunity for significant role conflict to arise, and even blaming of university education by students and graduates for creating a substantial level of role confusion (Lazarsfeld-Jensen, 2014).

Olsthoorn and Thompson (2018) identify that positive professional identity is an important component of entering a profession and should be considered in paramedic education. The influence that higher education has on what paramedics need to do during ambulance work is negligible – actions are determined by the needs of the patient. However, the knowledge, meta-cognitive skills and affective domains of competence that can be developed through a graduate-level education mean that the manner in which contemporary paramedics may conduct their ambulance practice may be significantly different from previous generations.

Perhaps the uncertainty relating to the importance of higher education for the formation of paramedic professional identity is reflected when exploring the transition from clinical practice into academia for paramedics (Munro et al., 2018). Academia is described as a ‘no-man’s land’ of professional identity (Munro et al., 2018). Even the choice of journal for this publication (*Nurse Education in Practice*) deepens the sense that outside of the ambulance environment, paramedic professional identity is uncertain and perhaps even incoherent for some paramedics. Indeed, Munro later found that paramedics working in academia no longer saw themselves as paramedics at all (Munro et al., 2019). This may say more about the nascence of graduate-level education for Australian paramedics than it does about paramedic professional identity per se, but it does serve to illustrate how fragile paramedic identity may be outside of ambulance practice in some cases, and for some people.

## Conclusion

Paramedic identity has developed from the history of the profession as stretcher bearers to the tertiary-educated healthcare professionals of today who have traditionally performed a unique role in emergency care in the ambulance service but who can be found working in a wide variety of different domains and areas.

Mounting levels of responsibility connected with a paramedic’s expanding scope of practice have resulted in many questioning how paramedics are professionally classified (Carter & Thompson, 2015). The approximate boundaries of the paramedic role in the United Kingdom are loosely found between statutory regulation (Health and Care Professions Council, 2018a, 2023), professional body assertions about scope of practice (College of Paramedics, 2021) and the experiences of diverse roles of practising paramedics (College of Paramedics, 2020, 2023). Given that any person’s identity is dynamic, nebulous, complex and heterogenous (Donnelly et al., 2015), it begs the question whether an overarching paramedic professional identity *can* be determined at all.

O’Meara (2011) argues that a theoretical framework that views professional practice in terms of ‘doing, knowing, being and becoming’ (Ewing & Smith, 2001) may help paramedicine develop its own professional philosophy. This would therefore suggest that paramedicine may be able to define its identity based on these aspects. This involves paramedics engaging in discourse, setting out what it is that paramedics do, why they do it in a particular way, what they know, their way of knowing, their way of becoming and their experience of ‘being’ a paramedic. Essentially, all paramedics must realise their potential as philosophers as well as clinicians, educators and researchers.

As the profession enters its third decade in the United Kingdom, what is needed is a cogent and consistent narrative of paramedicine. Grounding the profession in a coherent philosophy of paramedicine, which acknowledges paramedics’ perceptions of their professional identity, would provide a strong platform from which the profession can develop further. How paramedics negotiate their professional identity amid the shifting bergs of regulation, expectation and professional development, and indeed whether that identity persists outside of the ambulance environment, are areas worthy of future inquiry. A greater sense of how paramedics see themselves (being) may help to inform future paramedic students’ development (becoming), potentially avoiding some of the role conflict and tensions that can emerge when expectations arising from formative education experiences (knowing) are not met by the reality of practice (doing).

## Acknowledgements

Georgette Eaton would like to acknowledge Aidan Baron and Dr. Ruth Townsend for those discussions during autumn and winter 2020 and spring 2021 which gave gestalt to the development of this article.

## Author contributions

Both authors share joint authorship and have reviewed and approved the final version of the manuscript. LH acts as the guarantor for this article.

## Conflict of interest

None declared.

## Funding

Georgette Eaton acknowledges NHS Health Education England for its support of this article (ref: 190121). Georgette is also supported by a National Institute for Health Research (NIHR) Doctoral Research Fellowship (NIHR300681).
